# Dissociation of Immune Responses from Pathogen Colonization Supports Pattern Recognition in *C. elegans*

**DOI:** 10.1371/journal.pone.0035400

**Published:** 2012-04-13

**Authors:** Kwame Twumasi-Boateng, Michael Shapira

**Affiliations:** 1 Graduate Group in Microbiology, University of California, Berkeley, California, United States of America; 2 Department of Integrative Biology, University of California, Berkeley, California, United States of America; University of Birmingham, United Kingdom

## Abstract

*Caenorhabditis elegans* has been used for over a decade to characterize signaling cascades controlling innate immune responses. However, what initiates these responses in the worm has remained elusive. To gain a better understanding of the initiating events we delineated genome-wide immune responses to the bacterial pathogen *Pseudomonas aeruginosa* in worms heavily-colonized by the pathogen versus worms visibly not colonized. We found that infection responses in both groups were identical, suggesting that immune responses were not correlated with colonization and its associated damage. Quantitative RT-PCR measurements further showed that pathogen secreted factors were not able to induce an immune response, but exposure to a non-pathogenic *Pseudomonas* species was. These findings raise the possibility that the *C.elegans* immune response is initiated by recognition of microbe-associated molecular patterns. In the absence of orthologs of known pattern recognition receptors, *C. elegans* may rely on novel mechanisms, thus holding the potential to advance our understanding of evolutionarily conserved strategies for pathogen recognition.

## Introduction

The soil nematode *Caenorhabditis elegans* has been used for over a decade to study host-pathogen interactions. Such studies provided detailed information on pathogen-specific innate immune responses, and the signal transduction pathways and transcription factors that activated them (reviewed in [1]). However, what initiates innate immune responses in the worm remains unknown.

In vertebrates, innate immune responses are initiated mainly by recognition of pathogen-associated molecular patterns (PAMPs), such as lipopolysaccharide, peptidoglycan, or microbially-modified nucleic acids; PAMPs are occasionally referred to as MAMPs–microbe-associated molecular patterns, to acknowledge their existence in non-pathogenic microbes. In addition, recognition of damage-associated molecular patterns (DAMPs), such as ATP and monsodium urate crystals, can enhance activation by PAMPs, but also may be sufficient for initiation of innate immune responses [2]. The pattern recognition receptors (PRRs) responsible for recognizing PAMPs or DAMPs include members of several protein families including the Toll/Toll-like receptors (TLRs), NOD-like receptors (NLRs) and RIG-I-like nucleotide recognition receptors (RLRs) [3]. Innate immune recognition is conserved through evolution, both in terms of its general strategy as well as the proteins involved: The first TLR, Toll, originally identified as essential for *Drosophila's* development, was subsequently shown to also have a crucial role in innate immunity [4], [5], NLRs take part in antimicrobial defenses both in animals and in plants [6] and a RIG-I homolog was recently shown to function in *C. elegans* anti-viral defenses [7]. Although *C. elegans* mounts specific responses to infections with different pathogenic bacteria, manifested in a robust gene induction [8], it is yet unknown how it discriminates between different bacteria. *C. elegans* has one Toll homolog gene, *tol-1*, which appears to be is largely dispensable for immune protection ([9], [10], but see [11])).; Additionally, FSHR-1, a heterotrimeric G protein with leucine-rich repeats (a motif shared among all vertebrate TLRs and NLRs), is necessary for immune protection, but is equally protective against Gram-negative and–positive pathogens, suggesting that it might not function in the proximal events of pathogen recognition. [12]. The inability to identify *C. elegans* PRRs based on orthology raises the possibility that *C. elegans* may use novel modes of pathogen recognition. Alternatively, *C. elegans* may respond to pathogen-specific damage caused in the course of infection. To discriminate between these two possibilities we used an infection model of *C. elegans* intestinal colonization by *Pseudomonas aeruginosa* [13].

## Methods

### Strains

Worms were of the N2 wild-type strain. Bacterial strains included *E. coli* OP50-1, the clinical isolate *Pseudomonas aeruginosa* strain PA14, or a PA14 derivative expressing GFP off a stable plasmid [13]. *Pseudomonas mendocina*, a non-pathogenic environmental *Pseudomonad* was isolated from worms grown on soil (Montalvo-Katz, unpublished).

### Worm infection and sorting

Synchronized populations of wild-type worms, grown under standard conditions, were transferred at day two of adulthood either to *E. coli*, or to PA14-GFP. After eighteen hours, worms presented a wide-range of colonization reflected by accumulation of GFP-expressing bacteria in their intestine. Colonized (intensely green) and non-colonized (dark) worms were separated, either using the COPAS^TM^ BIOSORT worm sorter (Union Biometrica; two experiments) or by picking >100 worms of each group under a fluorescent stereoscope (one experiment, serving as a control for the automatic sorting).

### Testing the effects of the *P. aeruginosa* secretome on *C. elegans* immune responses

Three approaches were employed for testing potential contribution of *P. aeruginosa* secreted factors independently of the secreting bacteria; the results presented were obtained using the first and third of those: in the first approach, *P. aeruginosa* was grown on a 0.2 µM mixed cellulose esters filter (Millipore) placed on modified NGM plates at 37^°^C for 24 hours, at the end of which the underlying agar was blue due to secreted pyocyanin; the filter (containing bacteria) was then removed, *E. coli* added as food, and worms laid on plates; since filters may absorb some of the secreted molecules, particularly proteins, the second approach involved exposing worms to supernatants obtained from saturated *P. aeurigonsa* cultures; supernatants were cleared of bacteria by repeated centrifugation/transfer (microcentrifuge, 14 K RPM, 10 minutes each, 8 times), and following testing for absence of bacteria, 100 µl supernatant was added to lawns of dead *E. coli;* to account for molecules possibly secreted only on solid medium, in the third approach we submerged *P. aeruginosa* lawns in 1.2 ml M 9 solution, let it sit for an hour at room temperature to allow secreted factors to diffuse out of the agar and lawn, then collected supernatant, removed bacteria by repeated centrifugation as above, and added to lawns of dead *E. coli.* All three methods resulted in the same results.

### RNA extraction, microarrays and qRT-PCR

RNA was extracted from 100–700 worms per group/time-point using Trizol (Invitrogen). For microarray experiments, RNA was amplified using the MessageAmp™ II aRNA Amplification Kit (Ambion), labeled with the ULS™ aRNA Labeling Kit (Kreatech) and co-hybridized to Epoxy (Corning) microarrays spotted with 60-mer oligonucleotides (Washington University Genome Sequencing Center) with a similarly amplified and labeled reference RNA sample [14].

For (q)RT-PCR measurements, gene-specific threshold cycle (Ct) values were normalized to the respective actin values, and presented as fold change over the time = 0 point.

### PCR Primers

pan-actin forward TCGGTATGGGACAGAAGGAC

pan-actin reverse CATCCCAGTTGGTGACGATA

F55G11.2 forward TGGTTCTCCAGACGTGTTCA

F55G11.2 reverse CAGCCTTGCCTTTACTGACA

*lys-2* forward CCAATATCAAGCTGGCAAGG

*lys-2* reverse GTTGGATTGTTTGGCCAGTT

### Statistical analysis

Gene expression profiles obtained with microarrays were analyzed by a multi-class t-test using Significance Analysis of Microarrays (SAM; [15]), implemented as part of the TMEV software package. Based on T statistics the test retrieves genes with a T value above a cutoff score estimated to give the desired false discovery rate (selected to be 10%). This analysis was used to identify *C. elegans* genes differentially expressed in either one of the three analyzed groups: worms exposed to *E. coli*, worms exposed and colonized by *P. aeruginosa* PA14-GFP, or worms exposed to PA14-GFP, but not colonized. Since no difference was observed between expression profiles in worms collected manually or with the worm sorter, data from the three repeats for each of the three experimental groups were pooled. The resulting list of genes responding to PA14 contained 359 genes.

**Figure 1:**
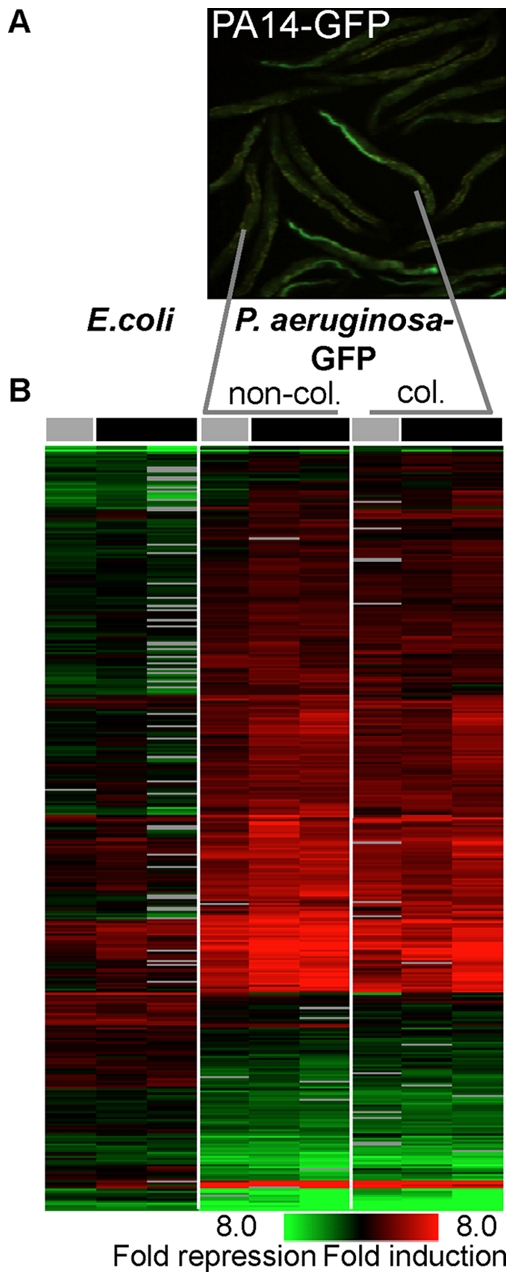
*C. elegans* immune responses to *Pseudomonas aeruginosa* are independent of colonization. A) 2-day old adult *C.elegans* exposed to GFP-expressing *P. aeruginosa* for 18 hrs show variability in colonization, allowing isolation of colonized and non-colonized worms. B) Gene expression profiles of *C. elegans* fed with *E. coli* or with GFP-expressing *P. aeruginosa* (colonized and non-colonized) for 18 hours; separation was achieved either by picking under a fluorescent stereoscope (1 experiment; grey bar), or using the COPAS^TM^ Worm Sorter (two independent experiments ; black bar). Shown are genes responding to the pathogen, as identified with a multi-class t-test analysis (10% false discovery rate).

## Results

The PA14 strain of *Pseudomonas aeruginosa*, made to express GFP (PA14-GFP from here on), can be followed as it colonizes the worm intestine, leading to death within three days. This colonization requires live bacteria and depends on bacterial regulators of virulence [16]. Furthermore, once reaching a significantly visible level of colonization (e.g. following 18 hours of exposure), most worms remain colonized, even when transferred to *E. coli* (65% of N = 96). This suggests active interactions between the pathogen and its host, which enable the pathogen to persist in most cases. When a genetically-homogenous and age-synchronized population of worms is exposed to PA14-GFP, significant heterogeneity is seen in colonization by the pathogen (Fig. 1A). We reasoned that if colonization-associated damage elicited immune responses in *C. elegans*, then immune responses would be correlated to the degree of colonization. To test this, we chose the extreme case of comparing significantly colonized worms with worms that were not visibly colonized. We exposed wild-type worms to PA14-GFP under standard conditions (or to *E. coli* control) and following 18 hours, separated significantly colonized worms (green) from visibly non-colonized (dark). Subsequently, gene expression was examined using microarrays in both groups as well as in those exposed to the *E. coli* control. Genes responding to *P. aeruginosa* were identified using a multi-class t-test with a false discovery rate of 10%. This analysis identified gene classes previously reported as being induced by *P. aeruginosa,* including lysozymes, lectins [14], [17], several neuropeptide-like genes, and detoxification genes [17] (Fig. 1B and Table S1). The analyzed dataset is attached as Table S2. Importantly, infection responses were largely independent of the degree of colonization. Thus, although worm death from PA14 infection is correlated with colonization, the responses against it were not.

**Figure 2:**
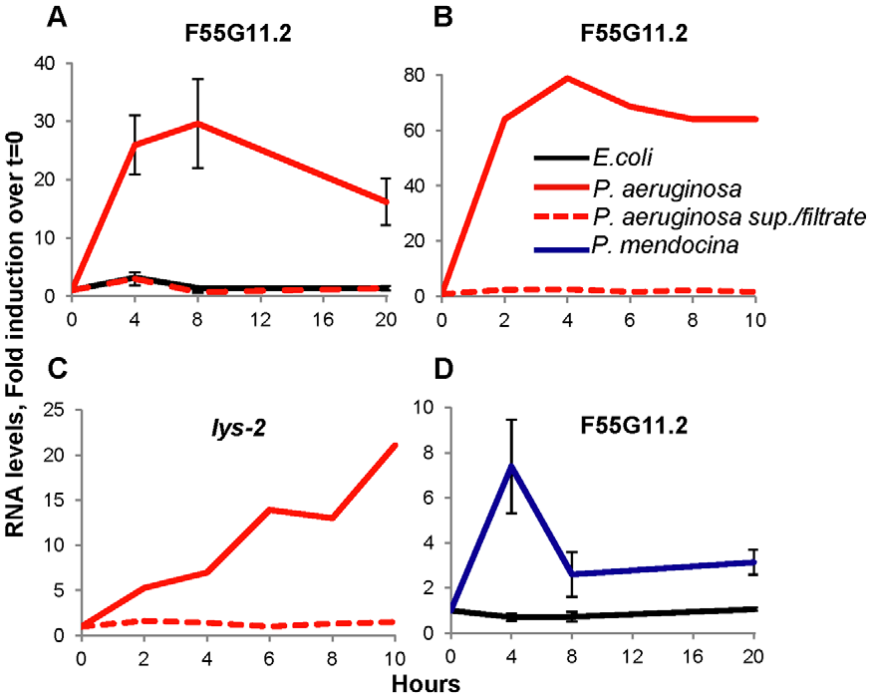
*Pseudomonas* secreted factors are not sufficient to induce immune responses, while conserved cell-associated factors are. Gene expression measured by qRT-PCR, following exposure of young-adult *C. elegans* (at T_0_) to *E. coli*, alone or with *P. aeruginosa* supernatant (A) or *P. aeruginosa* filtrate (B,C), or to the non-pathogenic *P. mendocina* (D).

Several possible explanations may account for immune responses without visible colonization. The first that we wished to be able to rule out was that worms that are not visibly colonized were previously colonized, but managed to clear the infection. To test this possibility under similar conditions as those of the experiment we picked visibly colonized worms (N = 80) following a 12-hour exposure to PA14-GFP, and continuing the exposure to PA14-GFP on new plates, we examined whether any of these worms appeared dark by the time of collection (6 hours later); this turned out not to be the case, as all worms remained green. This does not mean that ‘dark’ worms are completely non-colonized, as they may be colonized with an undetectably small number of bacteria, but the similarity between their immune responses and those of fully-colonized worms implies that the immune response is not correlated with the degree of colonization.

A second possibility is that molecules secreted by *P. aeruginosa* cause damage to the worm, which independent of colonization can induce the immune response. This is particularly plausible since *P. aeruginosa* is known to secrete a wide array of small-molecule and proteinaceous exotoxins [18]. To address this possibility, we used quantitative (q)RT-PCR to follow the expression of F55G11.2, a gene with a yet uncharacterized function, which was previously identified as part of the earliest responses to *P. aeruginosa* [14]. A closer examination showed that F55G11.2 was strongly induced within two hours of exposure to the pathogen (Fig. 2B) and as early as one hour following exposure (not shown). However, F55G11.2 was not induced when worms were exposed to a conditioned solution from a *P. aeruginosa* 24-hour culture (the ‘secretome’), laced onto dead *E. coli* (Fig. 2A). Induction of F55G11.2 was similarly missing when worms were grown on plates conditioned with *P. aeruginosa* grown on a filter, which was removed prior to transfer of worms and replaced with dead *E. coli* serving as food (Fig. 2B). Similar lack of induction was observed for *lys-2* and *pgp-5*, two additional infection response genes that respond to *P. aeruginosa*, but with a slower time course than F55G11.2 (Fig. 2C and results not shown). Thus, secreted factors are not sufficient to induce immune responses against *P. aeruginosa*.

A third possibility that we are unable to rule out is that volatile toxins released by *P. aeruginosa* cause damage to the worm, which induces immune responses. *P. aeruginosa* has a distinctive smell produced by a combination of volatile compounds. Of these, one is hydrogen cyanide, a potent toxin. However, cyanide production was not found to take part in PA14 pathogenicity in *C. elegans* (unlike the PA01 strain [19]) and without any additional known toxic volatile compounds released from *P. aeruginosa*, this is unlikely.

The fourth possible explanation for induction of immune responses prior to detectable colonization is that *C. elegans* can recognize molecules associated with the pathogen (i.e. PAMPs/MAMPs), with a sensitivity that allows it to respond to a small number of bacteria. The failure of the *P. aeruginosa* ‘secretome’ to induce immune responses, supported this possibility. To decouple structural features of *Pseudomonas* from its pathogenicity, we examined immune responses to *Pseudomonas mendocina,* a recently identified *C. elegans* commensal that shows no pathogenicity, both in terms of survival/lifespan as well as with regards to early symptoms of infection (i.e. muscle function and movement) (Fig. S1 and Montalvo-Katz, unpublished results). Exposure to intact *P. mendocina* lead to F55G11.2 gene induction, smaller than the response to *P. aeruginosa*, but reproducible (Fig. 2D). This is consistent with the hypothesis that *C. elegans* can recognize cell-associated moieties that are shared between *P. aeruginosa* and *P. mendocina,* and that such recognition plays a role in the initiation of early immune responses. Altogether, our data suggest that *C. elegans* immune responses against *P. aeruginosa* are initiated by PAMP recognition, or, since the recognized pattern is shared with the non-pathogenic *P. mendocina*, MAMP recognition.

## Discussion

We found that *C. elegans* immune response occurs prior to any visible colonization. Death, and presumably damage due to *P. aeruginosa* infection, is correlated with the extent of colonization. That this correlation does not hold for immune responses suggests sensitive detection of molecular patterns, apparently cell-associated and furthermore, shared among pathogenic and non-pathogenic *Pseudomonas* species. Recognition of MAMPs by *C. elegans* does not exclude the possibility that it can also respond to additional types of stimuli. In fact, the smaller magnitude of the response to *P. mendocina* compared to *P. aeruginosa* may be indicative of multiple signals, some cell-associated, but others associated with pathogenesis (perhaps DAMPs), leading to a full-blown immune response. The results described here provide evidence for MAMP recognition in *C. elegans*, but the nature of these MAMPs remains to be identified. Furthermore, since no PRR orthologs have been identified to date in *C. elegans*, these results further encourage a search for *C. elegans* PRRs, as they may represent novel mechanisms of pathogen recognition.

## Supporting Information

Figure S1 ***Pseudomonas mendocina*** **is a non-pathogenic species.** (A) Lifespan analysis of worms grown on *P. mendocina* shows comparable lifespan to that of worms grown on the normal food bacteria *E. coli* (N = 90–93 worms for each group). Differences between curves were evaluated statistically using Kaplan Meier survival analysis followed by the Logrank test (p = 0.3483). (B) Muscle function decline, represented by the rate of defecation, a coordinated muscle program [20], becomes apparent following 20 hours of exposure to the pathogen *P. aeruginosa*, but not to *E. coli* or *P. mendocina* (at 25^°^C). Dots represent average interval between defecations (n = 10 cycles, or less, when intervals exceeded four minutes) in individual young-adults; green bars represent medians. *p = 0.002 (t-test). The general speed of worm movement also decreased in *P. aeruginosa* but not in *P. mendocina* (not shown).
(TIF)

Table S1 Genes responding to *P. aeruginosa.*
(XLS)

Table S2 Full data set used for microarray analysis.
(XLS)
