# Supplementary figures and images for: Dissociation of Immune Responses from Pathogen Colonization Supports Pattern Recognition in C. elegans
Source: PLoS One. 2012 Apr 13;7(4):e35400. doi: 10.1371/journal.pone.0035400 (PMC3325959; doi:10.1371/journal.pone.0035400)

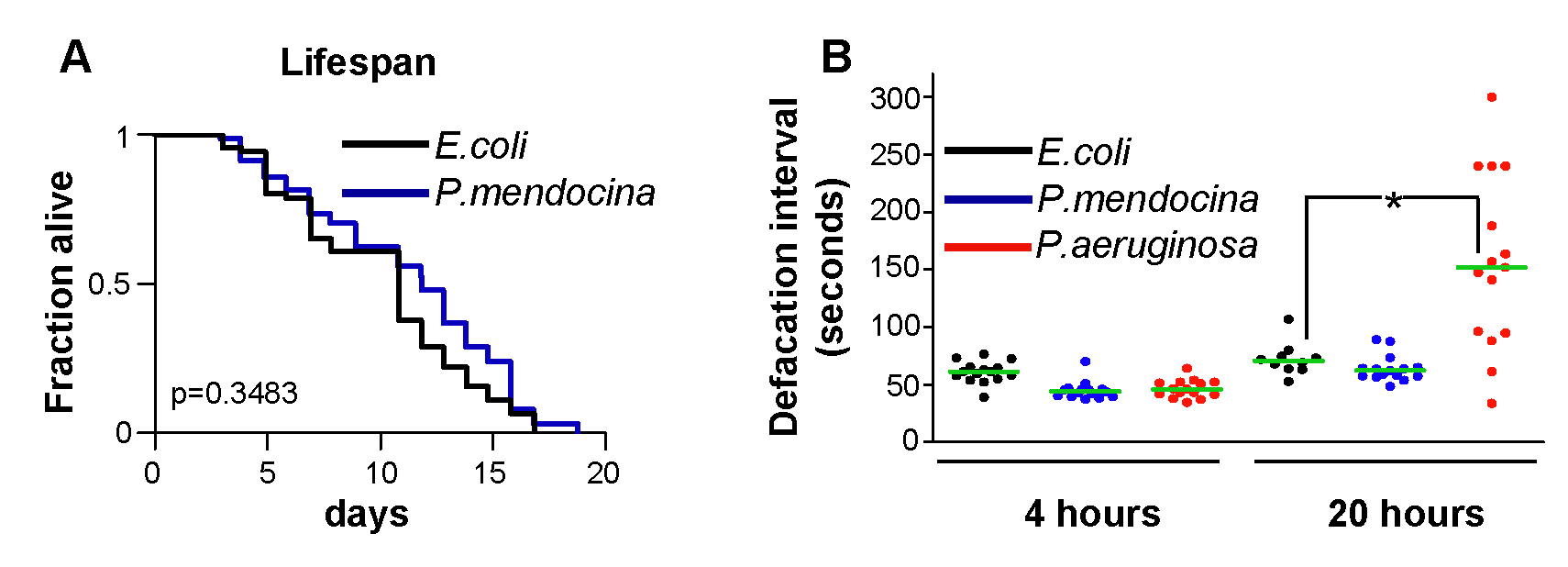

Supplement: Figure S1 — Pseudomonas mendocina is a non-pathogenic species. (A) Lifespan analysis of worms grown on P. mendocina shows comparable lifespan to that of worms grown on the normal food bacteria E. coli (N = 90–93 worms for each group). Differences between curves were evaluated statistically using Kaplan Meier survival analysis followed by the Logrank test (p = 0.3483). (B) Muscle function decline, represented by the rate of defecation, a coordinated muscle program [20], becomes apparent following 20 hours of exposure to the pathogen P. aeruginosa, but not to E. coli or P. mendocina (at 25°C). Dots represent average interval between defecations (n = 10 cycles, or less, when intervals exceeded four minutes) in individual young-adults; green bars represent medians. *p = 0.002 (t-test). The general speed of worm movement also decreased in P. aeruginosa but not in P. mendocina (not shown). (TIF) [file pone.0035400.s001.tif]
